# Clinical, microbiological, and molecular epidemiological characteristics of *Klebsiella pneumoniae*-induced pyogenic liver abscess in southeastern China

**DOI:** 10.1186/s13756-019-0615-2

**Published:** 2019-10-29

**Authors:** Siqin Zhang, Xiucai Zhang, Qing Wu, Xiangkuo Zheng, Guofeng Dong, Renchi Fang, Yizhi Zhang, Jianming Cao, Tieli Zhou

**Affiliations:** 1 Department of Clinical Laboratory, the First Affiliated Hospital of Wenzhou Medical University, Wenzhou, 325000 China; 2 School of Laboratory Medicine and Life Science, Wenzhou Medical University, Wenzhou, 325000 China

**Keywords:** Pyogenic liver abscess, Clinical characteristics, Hypervirulent *K. pneumoniae*, Virulence factors, Sequence type, Multidrug resistant

## Abstract

**Background:**

*Klebsiella pneumoniae*-induced pyogenic liver abscess (KP-PLA) has emerged as a life-threatening disease worldwide. However, to date, a limited number of scholars have attempted to systematically elucidate the characteristics of KP-PLA. The aim of the present study was to analyze clinical, microbiological, and molecular epidemiological characteristics of KP-PLA patients in Southeastern China.

**Methods:**

The KP-PLA cases from a tertiary teaching hospital in China from January 2016 to December 2017 were systemically studied and elucidated comprehensively. The virulence factors, resistant spectrum, and clones of *K. pneumoniae* isolates were identified with string test, polymerase chain reaction (PCR), antimicrobial susceptibility test, and multilocus sequence typing. Moreover, the characteristics in KP-PLA patients with and without other hepatobiliary diseases (OHD) were also been compared.

**Results:**

A total of 163 KP-PLA cases were enrolled, in which the majority of those cases were senior males, and often associated with multiple underlying diseases, including diabetes (49.7%). The remaining cases belonged to healthy individuals (50.3%). The clinical symptoms were common but nonspecific, characterized by increased inflammatory parameters and abnormal liver function parameters. The abscess was often right-sided solitary presentation (58.3%). Cephalosporin or carbapenem plus metronidazole combined with percutaneous puncture or catheter drainage were favorable therapeutics. Although low resistance rates of commonly used antimicrobial drugs (< 10%) were observed, twelve strains were identified as multidrug resistant (MDR) strains, and were mainly isolated from the OHD patients. The hypermucoviscosity, as well as K1 and K2 serotypes accounted for 30.7, 40.5, and 19.0%, respectively. Except for *iroN* (24.5%) and *magA* (45.4%), the high prevalence of virulence genes (e.g. *aerobactin*, *rmpA*, *mrkD*, *fimH*, *uge*, *ureA*, *entB*, *ybtA*, *kfuBC*, and *wcaG*) was identified (68.7–100.0%). Additionally, ST23 was found as a predominant sequence type (ST; 38.7%), and three novel STs (ST3507, ST3508 and ST3509) were noted as well.

**Conclusions:**

The present study reported the abundant hvKp strains in KP-PLA, as well as convergence of hypervirulent and MDR *K. pneumoniae* isolates from the KP-PLA patients, particularly those cases with OHD. Given the various clinical manifestations and destructive pathogenicity, determination of the comprehensive characteristics of such isolates is highly essential to effectively carry out for optimal management and treatment of KP-PLA.

## Background

Pyogenic liver abscess (PLA) is a potentially life-threatening suppurating infection of hepatic parenchyma disease, which was frequently observed worldwide [1–3]. The morbidity of PLA has remarkably increased in Asian regions, e.g. Taiwan, where the annual incidence has gradually increased from 10.83 to 15.45 cases per 100,000 population since 2000 to 2011, and the mortality is up to 2–19% [4, 5]. PLA is often concomitant with numerous underlying hepatobiliary diseases that may lead to a higher overall rate of bacterial colonization [6]. Presenting symptoms of PLA are multiple and low specific, including fever, right upper abdominal pain, vomiting, nausea, and asthenia, thereby indicating a major medical challenge in diagnosis and therapeutic management of PLA [4, 6].

In terms of causative pathogens, *Klebsiella pneumoniae* has found as a predominant pathogen, accounting for 50–88% of PLA patients who lived Asia during the past two decades [2, 7]. Notably, *K. pneumoniae* isolating from PLA mainly associates with hypervirulence, with distinct clinical manifestations, as well as phenotypic and genotypic characteristics [1]. In a recently conducted study, Ye et al. reported that 90.9% of the pathogens causing PLA were hypervirulent *K. pneumoniae* (hvKp) strains, and there were correlations between the incidences of PLA and high prevalence of hvKp strains [8, 9]. In contrast to classic *K. pneumoniae* (cKp), the emerging variant, which was first reported in Taiwan in 1986, exhibited hypermucoviscosity, unique capsular serotype, virulence gene, sequence type (ST) and resistant spectrum [3]. In addition, hvKp-induced PLA may occur in young and healthy individuals, and then, migrate to distant sites, thereby leading to extrahepatic complications, such as endophthalmitis, meningitis, and necrotizing fasciitis [1, 3]. To date, *K. pneumoniae*-induced pyogenic liver abscess (KP-PLA) has become a global destructive disease. Therefore, systemic investigations on the clinical and microbiological characteristics of recently emerged KP-PLA population are highly essential, for making a comparison with the previously reported ones.

The hypermucoviscosity confirmed by a positive string test is one of the major virulence factors of *K. pneumoniae* in PLA, and the isolates are mainly taken as hvKp strains into consideration. Moreover, *K. pneumoniae* strains isolated from PLA are often K1 or K2 capsular serotype [8, 9]. Capsule, consisting of polysaccharide termed K antigen, is associated with virulence. K1 and K2 serotype isolates are especially hypervirulent due to their capability to confer resistance to phagocytosis and intracellular killing by neutrophils in serum. In addition to these two serotypes, K5, K20, K54, and K57 capsular serotype isolates are also hypervirulent as well [10, 11]. Furthermore, multiple virulence genes regarded as severe virulence determinants of *K. pneumoniae* are highly prevalent in KP-PLA. In previous studies, *rmpA* was shown to act on synthesis of capsular polysaccharide in order to regulate mucoid phenotype [1, 12]. This gene was reported present in 87.5% of *K. pneumoniae* strains in the PLA [12]. Additionally, *aerobactin*, a dominant iron siderophore, was taken as a critical virulence factor into account in *K. pneumoniae*, as well as being further prevalent in KP-PLA [8, 13]. Moreover, it was found in 93–100% of hvKp strains, and could enhance the virulence by 100-fold according to the mouse lethality test. As a result, it was even regarded as a marker gene of hvKp [14]. Apart from these two genes, other virulence genes of *K. pneumoniae* isolates include *iroN*, *kfuBC*, *ybtA*, *entB* (encoding siderophores), *wcaG*, *magA*, *uge* (capsule-associated genes), *fimH* (encoding type 1 fimbriae), *mrkD* (encoding type 3 fimbriae), and *ureA* (encoding nitrogen) [15–18]. However, the prevalence of these multitudinous virulence factors in KP-PLA has not been systematically evaluated. Moreover, multilocus sequence typing (MLST) is advantageous for categorizing strains in molecular epidemiological studies, and ST23 has been found as the most common sequence type in KP-PLA [3, 16]. In addition, *K. pneumoniae* strains in PLA are sensitive to the majority of antimicrobial agents [19]. However, antibiotics-resistant hvKp strains have been reported by numerous studies [6, 19]. The convergence of virulence and resistance in isolates from KP-PLA is still little-known, which might potentially lead to a poor prognosis and even serious clinical crises.

Despite the growth of prevalence and strong pathogenicity of KP-PLA, a limited number of cases have been reported in China, and the existing ones only described a certain aspect of characteristics [16, 20]. In the present study, a retrospective study was carried out, which systemically investigated and comprehensively assessed the clinical, microbiological, and molecular epidemiological characteristics of 163 KP-PLA cases from a tertiary teaching hospital in Southeastern China from January 2016 to December 2017. Furthermore, we compared the characteristics between KP-PLA patients with and without other hepatobiliary diseases (OHD). The present study may contribute to improve our understanding on KP-PLA, and also provide significant insights for the development of further effective therapeutic strategies for clinical trials.

## Materials and methods

### Study subjects

This retrospective study was conducted on KP-PLA patients at the First Affiliated Hospital of Wenzhou Medical University (Wenzhou, China) from June 1, 2016 to December 31, 2017. This hospital is a 4100-bed major tertiary teaching hospital located in Southeast China (Wenzhou, China) with an annual admission of more than 160,000 inpatients. The diagnosis of KP-PLA was conducted based on the following criteria: (i) clinical features; (ii) imaging evidences; (iii) presence of *K. pneumoniae* in blood or pus culture; (iv) evidence of percutaneous puncture or surgical treatment, and (v) exclusion of amoebic, tuberculous liver abscess [21]. Then, the enrolled cases were divided into two groups: OHD and non-OHD patients. OHD included any hepatobiliary diseases except for PLA.

### Study data

The medical records were reviewed to integrally collect the data of KP-PLA patients during the study period. The study data included the following variables: demographic characteristics (age and gender), underlying or concomitant conditions (diabetes mellitus, biliary disease, hepatitis and cirrhosis, fatty liver, history of intra-abdominal trauma or surgery, malignancy, and hypertension), clinical symptoms, length of stay in hospital, admission temperature, admission to intensive care unit (ICU), imaging findings, laboratory values (C-reactive protein [CRP], white blood cell [WBC] count, red blood cell [RBC] count, alanine aminotransferase [ALT], aspartate aminotransferase [AST], albumin [ALB]), invasive procedure, details of therapeutic approach, drainage ways, abscess prognosis, and outcomes at discharge. The abscess prognosis was determined according to the criteria based on clinical symptoms and abscess changes, which were stipulated by the Chinese Academy of Medical Sciences.

### Clinical *K. pneumoniae* isolates

Initial strains were isolated from sterile fluid including pus, blood, and drainage fluid of KP-PLA patients and identified as *K. pneumoniae* by a matrix-assisted laser desorption/ionization time-of-flight mass spectrometry system. Antimicrobial susceptibility test for *K. pneumoniae* isolates was conducted by bioMerieux VITEK-2 (BioMérieux, Marcy-l’Étoile, France). The tested antibiotics included ampicillin, ampicillin/sulbactam, cefazolin, cefotetan, aztreonam, ceftriaxone, ceftazidime, cefepime, cefoperazone/sulbactam, ertapenem, imipenem, ciprofloxacin, levofloxacin, gentamicin, tobramycin, amikacin, sulfamethoxazole/trimethoprim, and nitrofurantoin. The results were interpreted by the latest guidelines published by the Clinical and Laboratory Standards Institute (CLSI; Pittsburgh, PA, USA). Multidrug resistant (MDR) strains were defined as non-susceptible to three or more different antimicrobial categories. Then, these strains were stored at − 80 °C for further research.

### String test

The bacterial colony of *K. pneumoniae* strains on the agar plate was stretched by an inoculation loop. The string test would be considered as positive, representing hypermucoviscosity when a strain generated a viscous string with a length of > 5 mm [22].

### Polymerase chain reaction (PCR) for capsular serotypes and virulence genes

Crude genomic DNA was extracted from *K. pneumoniae* strains. Subsequently, capsular serotype-specific genes (for serotypes of K1, K2, K5, K20, K54, and K57) and virulence genes (e.g. *aerobactin*, *rmpA*, *iroN*, *kfuBC*, *ybtA*, *entB*, *wcaG*, *magA*, *uge*, *fimH*, *mrkD*, *ureA*) were amplified by PCR using specific primes as previously described [15–18, 23, 24]. In addition, strains with these genes determined by PCR and DNA sequencing were selected as positive control for the subsequent PCR experiments.

### MLST

In the present study, seven housekeeping genes of *K. pneumoniae* (*gapA*, *mdh*, *phoE*, *tonB*, *infB*, *pgi*, and *rpoB*) were amplified and sequenced to characterize the genotypes of all isolates according to the provided protocols (http://bigsdb.pasteur.fr/klebsiella/klebsiella.html/). The alleles and STs were assigned according to the online database of the Pasteur Institute MLST for *K. pneumoniae*. In accordance with the genetic similarity diagram using the eBURSTv3 program, the clonal complexes (CCs) were analyzed to identify the molecular epidemiological relationships.

### Statistical analysis

All statistical analyses were performed using SPSS 22.0 software (IBM, Armonk, NY, USA). The categorical variables were listed as percentages and evaluated using the Chi-square test or Fisher’s exact test. The continuous data were expressed as mean ± standard deviation (mean ± SD) or median (25th - 75th percentile) appropriately and analyzed using the Student’s *t* test or Mann-Whitney *U* test. *P-*value < 0.05 was considered statistically significant. All tests were two-tailed.

## Results

### Clinical characteristics

The results showed that the annual morbidity of PLA was 17.68 to 20.62 cases per 10,000 inpatients since 2007 to 2018. From January 2016 to December 2017, a total of 163 KP-PLA cases that met the inclusion criteria were investigated in the current study. The clinical characteristics are summarized in Table 1. These cases were male-dominated (61.3%, 100/163) and had a median age of 63.0 (52.3–70.0) years old. The median length of stay in hospital was 17 (12–26) days. There were 82 cases (50.3%) in healthy individuals, while 81 cases (49.7%) had underlying or concomitant diseases, including 81 cases (49.7%) with diabetes mellitus, 46 cases (28.2%) with intra-abdominal trauma or surgery history, and 29 cases (17.8%) with OHD (i.e. hepatitis, cirrhosis, gallstones, choledocholithiasis, chronic cholecystitis or postcholecystectomy). The most common clinical symptoms were fever (84.0%, 137/163) and chill (81.0%, 132/163), with a median admission temperature of 38.9 (37.8–39.5) °C, followed by abdominal pain (39.9%, 65/163), frail (15.3%, 25/163), nausea or vomit (14.7%, 24/163), and abdominal distension (3.1%, 5/163). Additionally, four cases were found with metastatic infections, including endophthalmitis, cephalomeningitis, spontaneous bacterial peritonitis, and pneumonia, respectively.

**Table 1:** Clinical characteristics of *K. pneumoniae*-induced pyogenic liver abscess

Clinical characteristic	Value (*n* = 163)
Age, years	63.0 (52.3–70.0)
Gender	
Male	100 (61.3)
Female	63 (38.7)
Underlying or concomitant conditions	
Diabetes mellitus	81 (49.7)
History of intra-abdominal trauma or surgery	46 (28.2)
Hypertension	41 (25.2)
Malignancy	7 (4.3)
Biliary disease	23 (14.1)
Hepatitis and cirrhosis	8 (4.9)
Fatty liver	14 (8.6)
No underlying diseases	82 (50.3)
Admission temperature (°C)	38.9 (37.8–39.5)
Clinical Symptoms	
Fever (> 37.5 °C)	137 (84.0)
Chill	132 (81.0)
Abdominal pain	65 (39.9)
Vomit	24 (14.7)
Frail	25 (15.3)
Abdominal distension	5 (3.1)
Invasive procedure	141 (86.5)
Use of hormones and/or immunosuppressants	52 (31.9)
Multiplicity of the abscess	
Single	117 (71.8)
Multiple (≥2 abscesses)	46 (28.2)
Site of Single abscess	
Right hepatic lobe	95 (58.3)
Left hepatic lobe	22 (13.5)
Laboratory examination	
WBC count, × 10^9^/L	10.8 (7.9–13.4)
RBC count, × 10^12^/L	3.9 (3.5–4.2)
ALT(U/L)	39.0 (24.0–78.0)
AST(U/L)	37.0 (24.0–68.5)
Albumin, g/L	29.4 ± 5.1
Method of abscess treatment	
Simple antibacterial	30 (18.4)
Abscess drainage	129 (79.1)
Surgical removal	4 (2.5)
Prognosis of abscess	
Effective	116 (71.2)
Ineffective	47 (28.8)
Metastatic infections	
Endophthalmitis	1 (0.6)
Cephalomeningitis	1 (0.6)
Bacterial peritonitis	1 (0.6)
Pneumonia	1 (0.6)
Clinical outcomes	
Length of stay in hospital, days	17.0 (12.0–26.0)
Admission to ICU	16 (9.8)
Septic shock	15 (9.2)
Septicemia	20 (12.3)
In-hospital deaths	1 (0.6)

Values are presented as median (25th - 75th percentile), mean ± SD or No. (%) of patients

*WBC* White blood cell, *RBC* Red blood cell, *ALT* Alanine aminotransferase, *AST* Aspartate aminotransferase, *ICU* Intensive care unit

Medical imaging analysis revealed that solitary abscess accounted for 71.8% (117/163), of which 81.2% (95/117) were located in the right hepatic lobe, while multiple abscess only accounted for 28.2% (46/163). Significant laboratory abnormalities of inflammatory biomarkers and liver function indexes were observed, including increased CRP (100%, 163/163), WBC count (69.9%, 114/163), ALT (49.1%, 80/163), and AST (52.1%, 85/163). Moreover, serum hypoalbuminemia occurred in 96.9% (158/163) of cases, of whom, 43.0% (68/158) underwent severe serum hypoalbuminemia (Alb< 28 g/L).

In terms of clinical treatment, antibiotic therapy was applied to all the patients, 90.8% of cases (148/163) received a combination antibiotic therapy. The most common antimicrobial regimen was a third- or fourth-generation cephalosporin or carbapenem plus metronidazole. Drainage was implemented as the common treatment of abscess, in which 79.1% of cases (129/163) received percutaneous puncture or catheter drainage combined with antibiotic treatment, while 18.4% (30/163) received antibiotics-only treatment. The remaining four patients were surgically treated. However, there were 28.8% of patients (47/163) with ineffective prognosis of abscess. It is noteworthy that the majority of the patients with effective prognosis of abscess underwent drainage (*P* = 0.027), while the patients with ineffective prognosis of abscess mainly received antibiotics-only treatment (*P* = 0.005), as well as had the higher incidence of admission to ICU, septicemia, and septic shock. Overall, 9.2% of the patients (15/163) had septic shock, 12.3% of the patients (20/163) had septicemia, and 9.8% of the patients (16/163) were transferred to the ICU due to worsening condition, in which one case died during hospitalization.

### Microbiological and molecular epidemiological characteristics

Herein, *K. pneumoniae* strains were isolated from various clinical specimens, including pus (53.4%, 87/163), drainage fluid (24.5%, 40/163), blood (18.4%, 30/163), hydrothorax (1.2%, 2/163), and other sterile fluid (2.5%, 4/163). The microbiological characteristics of the 163 isolates are listed in Table 2. The majority of isolates were rarely resistant to commonly used antimicrobial drugs with low rates of resistance (< 10%), except for nitrofurantoin (20.9%), ampicillin/sulbactam (10.4%), and ampicillin (100%; intrinsic resistance). However, twelve isolates were still identified as MDR strains. In addition, two CRKP strains belonged to ST11 and were resistant to almost all antibiotics tested. Regrettably, the outcomes of patients isolated the CRKP strains were poor.

**Table 2:** Microbiological and molecular-epidemiological characteristics of *K. pneumoniae* strains isolated from KP-PLA patients

Characteristic	Value (*n* = 163)
Hypermucoviscosity	50 (30.7)
Capsular serotypes	
K1	66 (40.5)
K2	31 (19.0)
K5	8 (4.9)
K20	3 (1.8)
non-type	54 (33.1)
Virulence genes	
*aerobactin*	139 (85.3)
*rmpA*	155 (95.1)
*iroN*	40 (24.5)
*kfuBC*	116 (71.2)
*wcaG*	112 (68.7)
*ybtA*	120 (73.6)
*magA*	74 (45.4)
*fimH*	156 (95.7)
*mrkD*	163 (100.0)
*uge*	147 (90.2)
*entB*	144 (88.3)
*ureA*	145 (89.0)
Antimicrobial resistance	
Ampicillin	163 (100.0)
Ampicillin/sulbactam	17 (10.4)
Piperacillin/tazobactam	2 (1.2)
Cefazolin	10 (6.1)
Cefotetan	2 (1.2)
Aztreonam	5 (3.1)
Ceftriaxone	6 (3.7)
Ceftazidime	3 (1.8)
Cefepime	4 (2.5)
Cefoperazone/sulbactam	0 (0.0)
Ertapenem	3 (1.8)
Imipenem	5 (3.1)
Ciprofloxacin	5 (3.1)
Levofloxacin	4 (2.5)
Gentamicin	2 (1.2)
Tobramycin	2 (1.2)
Amikacin	1 (0.6)
SMZ-TMP	6 (3.7)
Nitrofurantoin	34 (20.9)
MDR strains	12 (7.4)
CRKP strains	6 (3.7)
Multilocus sequence typing	
ST23	63 (38.7)
ST29	10 (6.1)
ST65	14 (8.6)
ST86	10 (6.1)

Values are presented as No. (%) of isolates

*MDR* Multi-drug resistant, *CRKP* Carbapenems-resistant *K. pneumoniae, SMZ-TMP* Sulfamethoxazole and trimethoprim

**Fig. 1:**
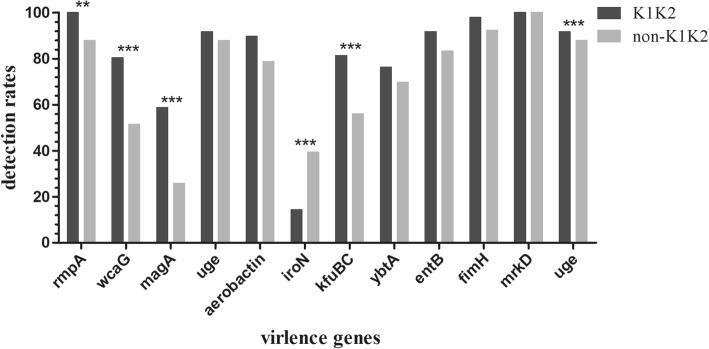
Comparing the detection rates of virulence genes between K1K2 strains and non-K1K2 strains. *, *P* < 0.05; **, *P* < 0.01; ***, *P* < 0.001

The string test disclosed that 30.7% (50/163) of strains exhibited hypermucoviscosity. As shown in Table 2, the dominant capsular serotype was serotype K1 (40.5%, 66/163), followed by serotype K2 (19.0%, 31/163), K5 (4.9%, 8/163), K20 (1.8%, 3/163), and non-type (33.1%, 54/163). Except for *iroN* and *magA*, all remaining virulence genes were presented in more than half of 163 strains. The prevalence of *rmpA* and *aerobactin* was up to 95.1 and 85.3%, respectively. Moreover, 88.0% (44/50) of hypermucoviscous *K. pneumoniae* isolates harbored *rmpA* and *aerobactin*, and 89.7% (87/97) of serotype K1K2 isolates possessed these two genes. Additionally, the prevalence of numerous virulence genes in the serotype K1K2 strains was notably higher than that in the non-K1K2 strains (Fig. 1).

**Fig. 2:**
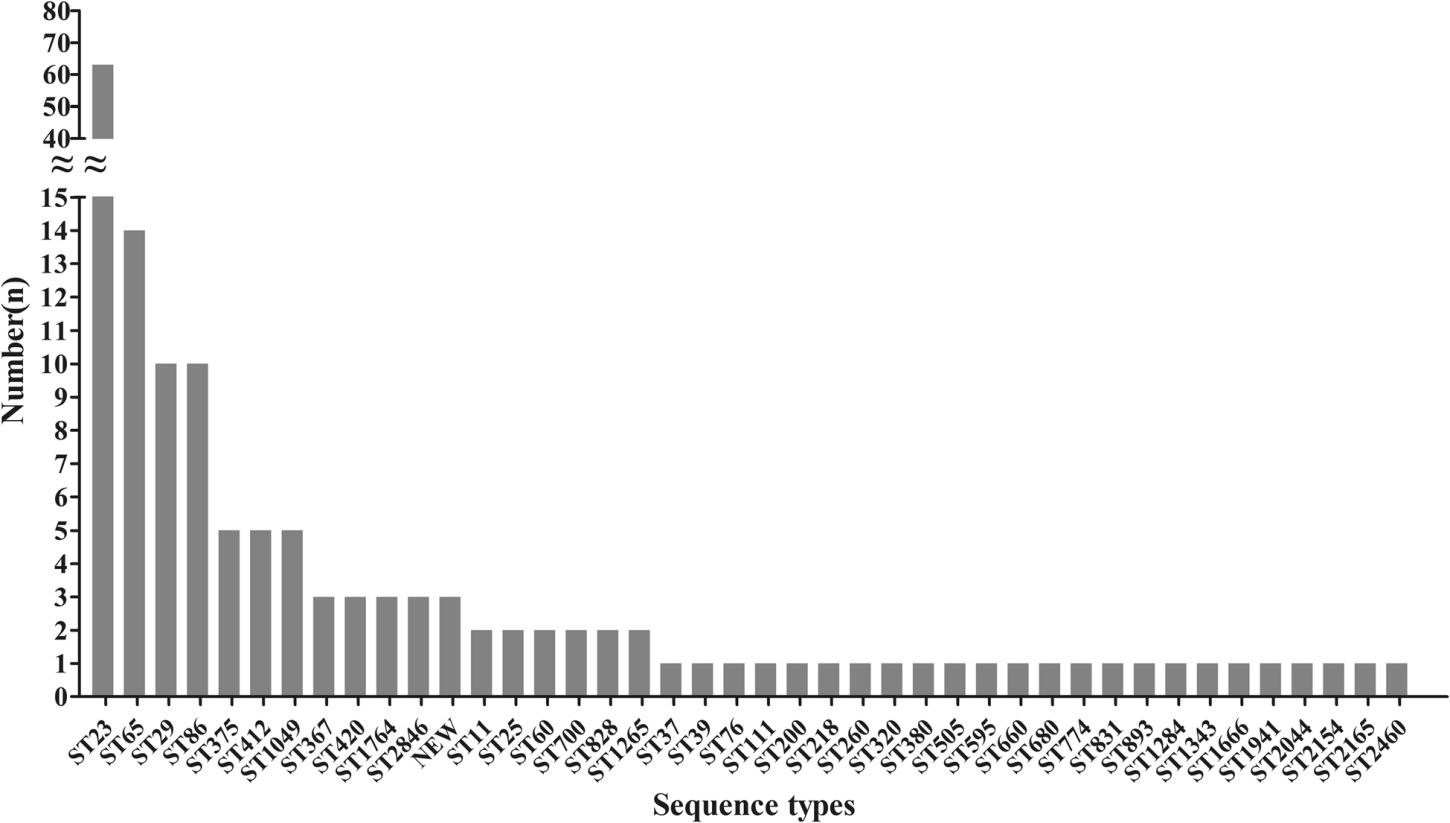
Specific types and quantities of all sequence types among the 163 *K. pneumoniae* strains

**Fig. 3:**
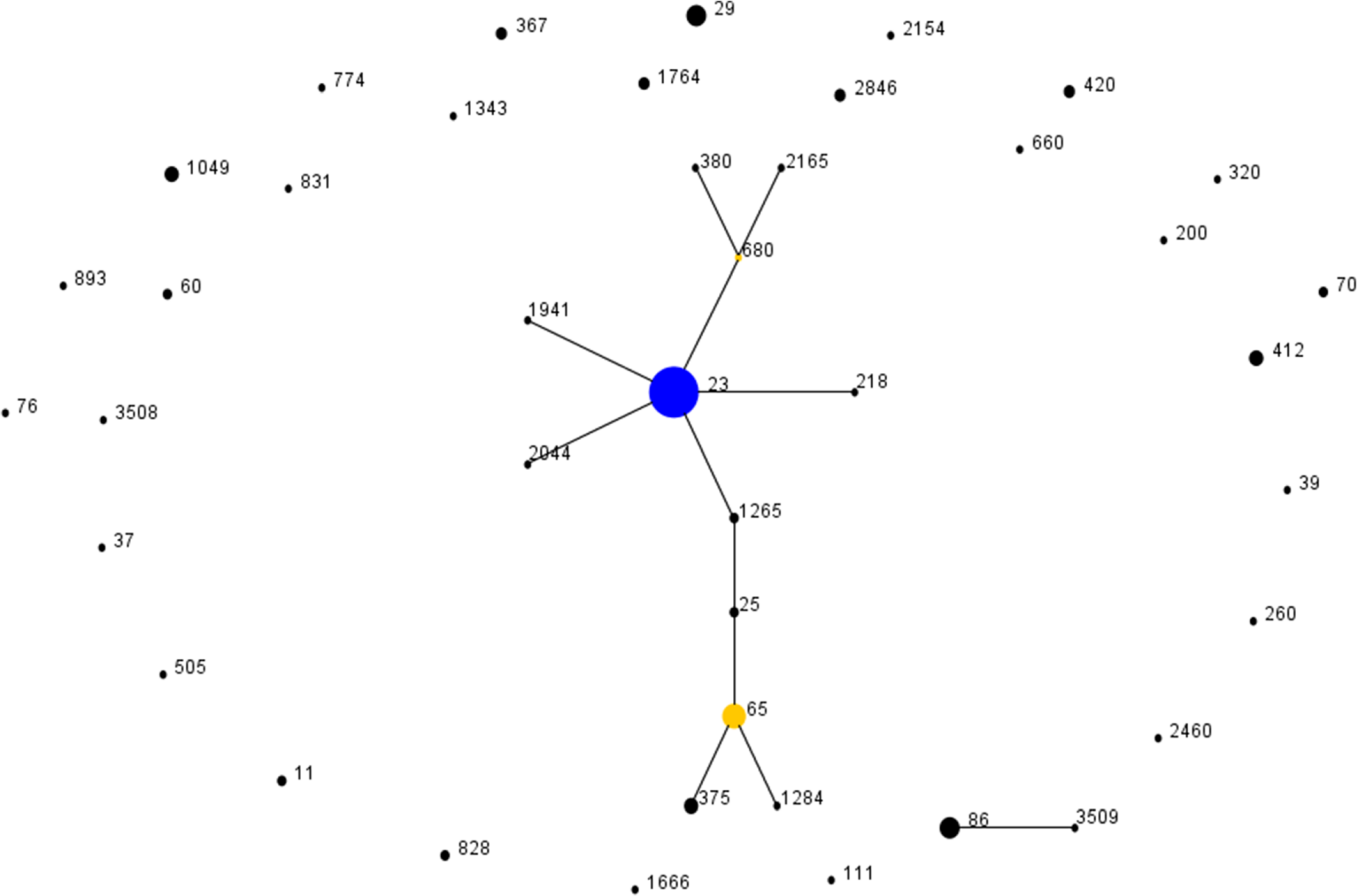
Performing eBURST analysis on molecular epidemiological characteristics of 163 *K. pneumoniae* isolates. The population snapshot indicates the clonal assignment of the STs presented in this study. Each black dot represents one ST, and blue dots indicate individual founders, while yellow spots denote sub-founders

Furthermore, MLST revealed 44 sequence types among the 163 *K. pneumoniae* strains, involving three newly identified STs (ST3507, ST3508, and ST3509). The predominant type was ST23 (38.7%, 63/163), followed by ST65 (8.6%, 14/163), ST29 (6.1%, 10/163), ST86 (6.1%, 10/163), and other STs (Fig. 2). Besides, eBURST analysis showed that those 44 STs were grouped into clonal complex (CC23, *n* = 1), doublet (D, *n* = 1) and singleton (S, *n* = 28) (Fig. 3). The CC23 included 57% (93/163) isolates covering 12 STs, and ST23 was defined as founder of the homologous complex. The D included ST86 and ST3509, in which those were only inconsonant with *pgi* allele. In addition, 69.8% (44/63) of ST23 strains belonged to K1 isolates, while 77.4% (24/31) of K2 isolates belonged to ST65- and ST86-like isolates.

### Comparing characteristics between KP-PLA patients with or without OHD

A total of 29 OHD patients were included in this study, while the remaining 134 cases were non-OHD patients. The differences in characteristics between these two groups are summarized in Tables 3 and 4. The median age was 67.0 (62.0–73.0) years old in the OHD group, which was older than that in the non-OHD group (*P* = 0.053). Furthermore, the OHD patients had a lower prevalence of diabetes (24.1% vs. 55.2%, *P* = 0.002) and more likely had the history of intra-abdominal trauma or surgery (89.7% vs. 14.9%, *P* = 0.000). Moreover, the length of stay in hospital was significantly longer in the OHD group than that in the non-OHD group (23 vs. 16 days, *P* = 0.045), and the clinical symptoms were inconspicuous. Notably, the prevalence of ST23 and *rmpA* in the OHD group was lower than that in the non-OHD group (*P* = 0.028, 0.049, respectively). On the contrary, the incidence of MDR strains in the OHD group was markedly higher than that in the non-OHD group (24.1% vs. 3.7%, *P* = 0.001). Therefore, the present study further analyzed the differences in resistant spectrum between these two groups. Surprisingly, the resistance rates of the majority of tested antimicrobials in the OHD group were remarkably higher than those in the non-OHD group, and the differences were statistically significant (Table 4).

**Table 3:** Comparing clinical characteristics between OHD patients and non-OHD patients from KP-PLA

Characteristic	OHD patients (*n* = 29)	non-OHD patients (*n* = 134)	*P* Value
Age, years	67.0 (62.0-73.0)	61.0 (51.0-70.0)	0.053
Gender			0.451
Male	16 (55.2)	84 (62.7)	
Female	13 (44.8)	50 (37.3)	
Underlying or concomitant conditions			
Diabetes mellitus	7 (24.1)	74 (55.2)	0.002*
History of intra-abdominal trauma or surgery	26 (89.7)	20 (14.9)	0.000*
Hypertension	5 (17.2)	45 (33.6)	0.084
Malignancy	2 (6.9)	5 (3.7)	0.797
Admission temperature (°C)	38.0 (37.3-39.0)	39.0 (38.0-39.5)	0.019*
Clinical Symptoms			
Fever (>37.5°C)	20 (69.0)	117 (87.3)	0.030*
Chill	23 (79.3)	109 (81.3)	0.800
Abdominal pain	14 (48.3)	51 (38.1)	0.308
Vomit	4 (13.8)	20 (14.9)	1.000
Frail	3 (10.3)	22 (16.4)	0.590
Abdominal distension	2 (6.9)	3 (2.2)	0.216
Invasive procedure	25 (86.2)	116 (86.6)	1.000
Use of hormones and/or immunosuppressants	11 (37.9)	41 (30.6)	0.442
Multiplicity of the abscess			0.409
Single	19 (65.5)	98 (73.1)	
Multiple (≥2 abscesses)	10 (34.5)	36 (26.9)	
Laboratory examination			
WBC count, ×10^9^/L	8.9 (5.9-11.6)	11.1 (8.2-14.0)	0.014*
RBC count, ×10^12^/L	3.9 (3.5-4.1)	3.9 (3.6-4.2)	0.667
ALT(U/L)	33.0 (14.0-49.0)	43.0 (27.0-82.8)	0.033*
AST(U/L)	35.0 (26.0-50.0)	37.0 (24.0-70.8)	0.621
Albumin, g/L	29.8±5.0	29.3±5.1	0.609
Method of abscess treatment			
Simple antibacterial	6 (20.7)	24 (17.9)	0.726
Abscess drainage	20 (69.0)	109 (81.3)	0.137
Surgical removal	3 (10.3)	1 (0.7)	0.021*
Prognosis of abscess			0.233
Effective	18 (62.1)	98 (73.1)	
Ineffective	11 (37.9)	36 (26.9)	
Metastatic infections			1.000
Endophthalmitis	0 (0.0)	1 (0.7)	
Cephalomeningitis	0 (0.0)	1 (0.7)	
Bacterial peritonitis	0 (0.0)	1 (0.7)	
Pneumonia	0 (0.0)	1 (0.7)	
Clinical outcomes			
Length of stay in hospital, days	23.0 (15.0-28.0)	16.0 (12.0-25.0)	0.045*
Admission to ICU	1 (3.4)	15 (11.2)	0.354
Septic shock	3 (10.3)	12 (9.0)	1.000
Septicemia	5 (17.2)	15 (11.2)	0.557
In-hospital deaths	0 (0.0)	1 (0.7)	1.000

Values are presented as median (25th - 75th percentile), mean ± SD or No. (%) of patients

*WBC* white blood cell, *RBC* red blood cell, *ALT* alanine aminotransferase, *AST* aspartate aminotransferase, *ICU* intensive care unit *P < 0.05

**Table 4:** Comparing microbiological and molecular-epidemiological characteristics of *K. pneumoniae* strains between OHD patients and non-OHD patients

Characteristic	Kp from OHD patients (*n* = 29)	Kp from non-OHD patients (*n* = 134)	*P* Value
Hypermucoviscosity	8 (27.6)	42 (31.3)	0.691
Capsular serotypes			
K1	11 (37.9)	55 (41.0)	0.757
K2	5 (17.2)	26 (19.4)	0.788
K5	1 (3.4)	7 (5.2)	1.000
K20	1 (3.4)	2 (1.5)	0.447
non-type	11 (37.9)	43 (32.1)	0.545
Virulence genes			
*aerobactin*	22 (75.9)	117 (87.3)	0.197
*rmpA*	25 (86.2)	130 (97.0)	0.049*
*iroN*	4 (13.8)	36 (26.9)	0.138
*kfuBC*	17 (58.6)	99 (73.9)	0.100
*wcaG*	18 (62.1)	94 (70.1)	0.395
*ybtA*	23 (79.3)	97 (72.4)	0.443
*magA*	9 (31.0)	65 (48.5)	0.087
*fimH*	29 (100.0)	127 (94.8)	0.451
*mrkD*	29 (100.0)	134 (100.0)	NA
*uge*	26 (89.7)	121 (90.3)	1.000
*entB*	24 (82.8)	120 (89.6)	0.475
*ureA*	27 (93.1)	118 (88.1)	0.646
Antimicrobial resistance			
Ampicillin	29 (100.0)	134 (100.0)	NA
Ampicillin/sulbactam	7 (24.1)	10 (7.5)	0.020*
Piperacillin/tazobactam	2 (6.9)	0 (0.0)	0.031*
Cefazolin	6 (20.7)	4 (3.0)	0.001*
Cefotetan	2 (6.9)	0 (0.0)	0.031*
Aztreonam	5 (17.2)	0 (0.0)	0.000*
Ceftriaxone	5 (17.2)	1 (0.7)	0.000*
Ceftazidime	3 (10.3)	0 (0.0)	0.005*
Cefepime	4 (13.8)	0 (0.0)	0.001*
Cefoperazone/sulbactam	0 (0.0)	0 (0.0)	NA
Ertapenem	2 (6.9)	1 (0.7)	0.082
Imipenem	3 (10.3)	2 (1.5)	0.040*
Ciprofloxacin	5 (17.2)	0 (0.0)	0.000*
Levofloxacin	4 (13.8)	0 (0.0)	0.001*
Gentamicin	1 (3.4)	1 (0.7)	0.325
Tobramycin	1 (3.4)	1 (0.7)	0.325
Amikacin	0 (0.0)	1 (0.7)	1.000
SMZ-TMP	4 (13.8)	2 (1.5)	0.008*
Nitrofurantoin	13 (44.8)	21 (15.7)	0.000*
MDR strains	7 (24.1)	5 (3.7)	0.001*
CRKP strains	3 (10.3)	3 (2.2)	0.119
Multilocus sequence typing			
ST23	6 (20.7)	57 (42.5)	0.028*
ST29	3 (10.3)	7 (5.2)	0.538
ST65	2 (6.9)	12 (9.0)	1.000
ST86	2 (6.9)	8 (6.0)	1.000

Values are presented as No. (%) of isolates

*MDR* Multi-drug resistant, *CRKP* Carbapenems-resistant *K. pneumoniae, SMZ-TMP* sulfamethoxazole and trimethoprim, *NA* not applicable, *P < 0.05

## Discussion

The incidence of PLA has notably elevated in recent years and accordingly, *K. pneumoniae* emerged as a life-threatening bacterial pathogen across Asian and European countries, as well as the United States [3, 25]. To our knowledge, KP-PLA has still remained as a mortality-associated infectious disease worldwide [3, 25]. To date, a limited number of studies have concentrated on characterization of the clinical and pathogenic features from one KP-PLA population simultaneously. Hence, in the present retrospective study, we systematically analyzed the 163 KP-PLA cases in a tertiary teaching hospital in Southeast China, which made the parallel comparability of clinical and microbiological data, and further supplemented the current data of KP-PLA worldwide. Moreover, we compared the characteristics between KP-PLA patients with or without OHD. To the best of our knowledge, this study was the first systematic analysis on these two groups of KP-PLA.

The data of annual morbidity in the current investigation confirmed high incidence of PLA in the last decade. Male gender, patients with diabetes, OHD and intra-abdominal trauma or surgery were more susceptible to have KP-PLA, which was comparable with the latest epidemiological trends [5–7]. Diabetes is known as an important risk factor for KP-PLA. A poor glycemic control could impair the neutrophil phagocytosis and promote growth of pathogen in tissues, while metabolic disorders could negatively influence the liver [21, 26]. Therefore, the blood sugar level should be strictly monitored and controlled for KP-PLA patients. Additionally, the KP-PLA patients with OHD or intra-abdominal trauma or surgery were not uncommon in the present study. A possible explanation was that *K. pneumoniae* easily entered into the liver via direct spreading, portal circulation or cross the intestinal barrier to cause KP-PLA and severe *K. pneumoniae* infection in those patients [27–29]. It indicated that such patients should be strictly followed-up and reviewed regularly in order to detect any possible signs and treat KP-PLA in time. In addition, about half of the cases occurred in healthy individuals, demonstrating a strong possibility of having an invasive fatal PLA caused by hvKp [1, 8]. The clinical manifestations and imaging findings of 163 KP-PLA cases were consistent with previous studies [8, 9, 20, 21]. Furthermore, the laboratory outcomes revealed that the majority of patients underwent inflammation and impaired liver function. However, the definite diagnosis of KP-PLA is often delayed due to lack of typical symptoms, and further progressed to fearful septicemia or septic shock [29]. It indicated that physicians are encouraged to make a high clinical suspicion on KP-PLA during presentation of the above-mentioned signs.

For patients who diagnosed with KP-PLA, percutaneous drainage under ultrasonography or computed tomography guidance combined with a proper antimicrobial therapy has been taken as a standard treatment into consideration [30]. The data of drainage rate and abscess prognosis existing in the current study re-verified the necessity of drainage for KP-PLA patients. Drainage may contribute to a better control of infection source, accurate identification of pathogens, and rational application of antibiotics. Although fatal metastatic infections were seldom observed in the present investigation, those extrahepatic invasive complications, especially in eye, lung or central nervous system, were required to be observed prudently for site-directed treatment in-time. The in-hospital mortality rate (0.6%) in the present investigation was lower than a previous study (5%) [1], which may be due to accurate and timely implementation of interventions for KP-PLA. In addition, a fraction of patients with serious conditions gave up treatment, it may lead to underestimate the mortality rate. Taken together, it is imperative for KP-PLA patients to perform a reasonable management in advance to attenuate morbidity, complications, and mortality.

In terms of microbiological characteristics of isolates from KP-PLA, virulence and antibiotic resistance were considered to play a substantial role in bacterial pathogenesis. The hypermucoviscosity was considered as a surrogate marker for hvKp and a significant contributor to the virulence for invasive KP-PLA infections [1, 3]. However, only a third of isolates possessed hypermucoviscosity in the present research. The rate of hypermucoviscosity was compatible with another recent study conducted in East China [20], while that was remarkably lower than that reported in other Asian countries (more than 70%) [3, 16, 19]. The majority of recently conducted studies suggested that the correspondence between hypermucoviscosity and hypervirulence was variable, and therefore, the virulence of isolates from KP-PLA should be assessed by a combination of genotypic and clinical features [22]. A polysaccharide capsule acts as a major virulence factor for hvKp by protecting *K. pneumoniae* from phagocytosis of immune cells and bactericidal action of complement or antimicrobial peptides [10]. *K. pneumoniae* strains are presented in at least 78 capsular serotypes, in which K1 and K2 are related to hvKp, as well as being pathogenic to humans strongly [11]. In the present research, the prevalence of K1 and K2 was 40.5 and 19.0%, respectively, suggesting that substantial percentages of isolates from KP-PLA were hypervirulent. Compared to previous reports (K1 ranged from 46.6 to 63.4%, and K2 ranged from 14.2 to 20.5%) [16, 31], it needs to be characterized whether geographical differences accounted for this distinction. Meanwhile, the non-K1K2 isolates also played a pivotal role in KP-PLA and should not be overlooked because these isolates may be hypervirulent as well.

To rule out one-sidedness in assessment of hypervirulence based on hypermucoviscosity or K1K2 serotypes [3, 19], the virulence genes of all the 163 strains were tested. Thereinto, *rmpA* and *aerobactin* are the most important genes for hypervirulence. *rmpA* regulates the synthesis of extracellular polysaccharide capsule and is responsible for hypermucoviscosity, the ablation of *rmpA* may lead to the loss or thinning of capsule, thereby weakening the ability to evade immune responses, in turn causing virulence of *K. pneumoniae* to be markedly reduced [12, 14]. The high prevalence of *rmpA* (95.1%) was observed in the current research. However, a remarkable number of *rmpA-*positive strains did not show hypermucoviscosity, reflecting that there may be other regulatory mechanisms for expression of hypermucoviscosity. For instance, *wcaG*, *magA*, and *uge* genes associate with biosynthesis of the capsule. These genes were also found to be prevalent in KP-PLA isolates. Another important virulence gene is *aerobactin*, which is essential for the growth and virulence of *K pneumoniae* in the host via regulation of iron supply. The *aerobactin*-positive strains were up to 85.3% in the current study. In addition, *aerobactin* and *rmpA* are often concomitant with hypermucoviscosity and have been extensively used to define hvKp [13, 32, 33]. In the study, 84.0% of isolates simultaneously carried *aerobactin* and *rmpA*, which were more likely to be hvKp strains, while did not always belong to K1K2 strains or possessed hypermucoviscosity. These observations were consistent with findings of previous studies [8, 32]. Other siderophores genes (*iroN*, *kfuBC*, *ybtA* and *entB*), mediating uptake of ferric iron were reported as virulence genes in KP-PLA [13, 15, 18]. In addition to *iroN*, the other three siderophores genes were possessed by the majority of KP-PLA isolates. Furthermore, *fimH* and *mrkD* mediate adhesion through encoding *K. pneumoniae* type 1 and type 3 fimbriae, and *mrkD* may promote development of biofilms. Besides, *ureA*, an α-subunit of the urease, is associated with invasion [18, 34]. In the present study, *fimH* and *mrkD* were observed in almost all isolates except for seven *fimH-*negative strains, and 89.0% of isolates possessed *ureA.* Therefore, clinicians are advised to consider phenotypes and genotypes of pathogen, and also carefully select an appropriate catheter and more frequent catheter irrigation or replacement to reduce bacterial adhesion and colonization. Altogether, all the 163 isolates harbored various virulence genes, reflecting that the majority of *K. pneumoniae* isolates from PLA exhibited hypervirulence and strong pathogenicity. Additionally, the majority of virulence genes, as well as the hypermucoviscosity, were notably widespread in K1K2 strains, suggesting that K1K2 isolates from KP-PLA typically carried various hypervirulent factors.

Although virulence factors did not always singularly determine hvKp, certain intrinsic correlations were noted, which required further investigation and characterization. In particular, virulence factors acted as a warning of hvKp-PLA, which recommended prolonged treatment properly and long-term follow-up to maximize the possibility of a successful treatment and minimize relapse rate [19, 22]. Regrettably, the current study could not present accurate numbers of hvKp in the 163 KP-PLA cases due to the fact that a reference standard for hvKp has not been defined yet [19, 22]. Nevertheless, from the microbiological perspective, clinicians are recommended to do not ignore the detection value of these virulence factors in KP-PLA isolates, especially after combining with antibiotic resistance. Meanwhile, the exploration of virulence factors may provide novel therapeutic targets and develop novel vaccines for hvKp infections, which are not limited to only the management of KP-PLA [35].

Additionally, *K. pneumoniae* isolates from KP-PLA were highly susceptible to almost all kinds of antimicrobial agents, such as β-lactamase inhibitors, third generation cephalosporins, and carbapenems, which might be related to abundant hvKp in KP-PLA [1, 36]. Nevertheless, twelve MDR strains were detected in the present study, which rarely occurred in KP-PLA [8], and these MDR strains were more likely to occur in the OHD patients. Further analysis revealed that the resistance rates of almost all tested antibiotics in the OHD group were significantly higher than those in non-OHD group. These results indicated that the accelerated emergence of resistance and colonization could be induced by increasing hepatobiliary interventions with routine prophylactic antibiotics in such patients [6]. However, it also could not be neglected to prevent infections for those patients because of unimaginable outcomes once hvKp occurred with MDR. Moreover, two ST11 CRKP isolates from the current research simultaneously disclosed hypervirulence, extensively drug resistance, and terrible pathogenicity, highlighting the significant threat of such real superbugs. An emerged clinical challenge is the convergence of hypervirulence and extensively drug- and pan-drug-resistant in *K. pneumoniae*, which might lead to further emergence of a “post-antibiotic” scenario [37]. These findings demonstrated that clinicians should be highly prudent and cautious in prescribing antibiotics and dosage options, in which host, pathogen, and host-pathogen interactions need to be taken into account to prevent MDR-hvKp, especially in the OHD patients.

MLST analysis uncovered the molecular epidemiological characteristics of *K. pneumoniae* strains in the 163 KP-PLA cases. Additionally, ST23 was found as a predominant type of KP-PLA with a rate of 38.7%, which was similar to a previous report [16]. ST23 is one of the dominant clones of hvKp and defined as a founder of clonal lineage CC23, representing a specific genetic background that conferred hypervirulence and fitness [16, 19, 37]. Even though CC23 has a capacity to spread worldwide through multiple international transmission events rather than local expansions, CC23 isolates from KP-PLA were genotypically closely related [38]. It is noteworthy that ST23 was firmly linked to serotype K1, while ST65- and ST86-like isolates were associated with serotype K2 in KP-PLA as similar to other reports performed in Asia [20, 25], while the reason has still remained elusive. Those findings disclosed strict association of hypervirulent clones with capsular serotypes. Meanwhile, there still existed 43% of strains that belonged to clones other than CC23, including three novel sequence types. The novel ST3509 was a single locus variant of ST86, which expanded the cloned lineage of CC86. The existence of doublets and singletons demonstrated the genetic diversity in isolates from KP-PLA.

However, the current study contains a number of limitations. Firstly, it was designed as a retrospective study at a single center, that might increase selection and information biases. Secondly, for reasons already explained, no accurate data of hvKp were reported. Further in vitro and in vivo virulence-based experiments may be required for isolates from KP-PLA, such as galleria mellonella models, mice models, and neutrophil phagocytosis assay.

## Conclusions

The present study may draw attention from clinicians to take into account KP-PLA in patients with non-specific symptoms, and indicate the necessity to fully combine the auxiliary examination for early diagnosis and appropriate treatment, in order to improve outcome and prevent severe metastatic complications. Management of the underlying diseases is decisive in KP-PLA patients, especially in OHD patients. There existed large quantities of hvKp in KP-PLA, and even MDR-hvKp. With the prospective convergence of virulence and resistance, it is essential to perform prompt identification of characteristics of *K. pneumoniae*, followed by effective therapeutic strategies. Hence, additional studies need to be conducted to elucidate the relationships between the phenotypes, genotypes, resistant spectrum and clones of *K. pneumoniae* isolates from KP-PLA, with specific focus on hypervirulence, MDR, and pathogenicity. Further researches can be helpful to raise the awareness of hvKp and provide effective treatments for KP-PLA patients.

## Data Availability

All data generated or analysed during this study are included in this manuscript.

## Abbreviations

ALB: Albumin
ALT: Alanine aminotransferase
AST: Aspartate aminotransferase
CCs: Clonal complexes
cKp: classic *K. pneumoniae*
CLSI: Clinical and Laboratory Standards Institute
CRP: C-reactive protein
hvKp: hypervirulent *K. pneumoniae*
ICU: Intensive care unit
KP-PLA: *Klebsiella pneumoniae*-induced pyogenic liver abscess
MDR: Multidrug resistant
MLST: Multilocus sequence typing
OHD: Other hepatobiliary diseases
PCR: Polymerase chain reaction
PLA: Pyogenic liver abscess
RBC: Red blood cell
STs: Sequence types
WBC: White blood cell
